# Interplay between rhizospheric *Pseudomonas chlororaphis* strains lays the basis for beneficial bacterial consortia

**DOI:** 10.3389/fpls.2022.1063182

**Published:** 2022-12-15

**Authors:** Rafael Villar-Moreno, Sandra Tienda, Jose A. Gutiérrez-Barranquero, Víctor J. Carrión, Antonio de Vicente, Francisco M. Cazorla, Eva Arrebola

**Affiliations:** ^1^ Mango and Avocado Microbiology Group, Department of Microbiology, Faculty of Sciences, University of Málaga, Málaga, Spain; ^2^ Department of Microbiology and Plant Protection, Instituto de Hortofruticultura Subtropical y Mediterránea “La Mayora”, IHSM-UMA-CSIC, Málaga, Spain

**Keywords:** *Pseudomonas chlororaphis*, competitive index, avocado, rhizosphere, root colonization, syncom, consortia

## Abstract

*Pseudomonas chlororaphis* (Pc) representatives are found as part of the rhizosphere-associated microbiome, and different rhizospheric Pc strains frequently perform beneficial activities for the plant. In this study we described the interactions between the rhizospheric Pc strains PCL1601, PCL1606 and PCL1607 with a focus on their effects on root performance. Differences among the three rhizospheric Pc strains selected were first observed in phylogenetic studies and confirmed by genome analysis, which showed variation in the presence of genes related to antifungal compounds or siderophore production, among others. Observation of the interactions among these strains under lab conditions revealed that PCL1606 has a better adaptation to environments rich in nutrients, and forms biofilms. Interaction experiments on plant roots confirmed the role of the different phenotypes in their lifestyle. The PCL1606 strain was the best adapted to the habitat of avocado roots, and PCL1607 was the least, and disappeared from the plant root scenario after a few days of interaction. These results confirm that 2 out 3 rhizospheric Pc strains were fully compatible (PCL1601 and PCL1606), efficiently colonizing avocado roots and showing biocontrol activity against the fungal pathogen *Rosellinia necatrix*. The third strain (PCL1607) has colonizing abilities when it is alone on the root but displayed difficulties under the competition scenario, and did not cause deleterious effects on the other Pc competitors when they were present. These results suggest that strains PCL1601 and PCL1606 are very well adapted to the avocado root environment and could constitute a basis for constructing a more complex beneficial microbial synthetic community associated with avocado plant roots.

## 1 Introduction

Currently, the scientific community is very conscious of the influence and importance of the microorganisms inhabiting plants, known as plant microbiota (Chialva et al., 2022). This microbial community is fundamental for plant fitness, and it is common to find beneficial groups of bacteria, especially those related to root disease suppression, that can be actively recruited by the plants, thus forming what is known as the holobiont and contributing to the phenomenon called “indirect defence” or “plants crying for help” (Vandenkoornhuyse et al., 2015; Berendsen et al., 2018; Rolfe et al., 2019; de la Fuente Cantó et al., 2020; Trivedi et al., 2020). Some of these beneficial microbial members from these plant-related rhizobacterial communities usually display activities that are positive for plant health (Raaijmakers et al., 2009), such as plant growth promotion (PGP) activities (Spaepen and Vanderleyden, 2011; Sarma et al., 2015; Singh et al., 2015; Arrebola et al., 2019; Hestrin et al., 2019).

Proteobacteria is one of the most abundant phyla in bacterial communities associated with plant roots, followed by Firmicutes, Bacteroidetes and Actinobacteria (Trivedi et al., 2020). The genus *Pseudomonas* belongs to Proteobacteria and comprises almost 300 different species (Parte, 2014; Garrido-Sanz et al., 2016). This genus includes several soil- and root-associated species displaying PGP activities (Mercado-Blanco and Bakker, 2007), which are mainly included in the *Pseudomonas fluorescens* complex, which can be divided into nine different phylogenetic groups based on multilocus sequence analysis of type strains: *P. fluorescens, P. gessardii, P. fragi, P. mandelii, P. jessenii, P. koreensis, P. corrugata, P. protegens and P. chlororaphis* (Garrido-Sanz et al., 2016). Currently, the phylogenetic group of *P. chlororaphis* contains four different subspecies: *P. chlororaphis* subsp. *chlororaphis, P. chlororaphis* subsp. *aurantiaca, P. chlororaphis* subsp*. piscium and P. chlororaphis* subsp*. aureofaciens*. Many strains belonging to the *P. chlororaphis* phylogenetic group have been proven to be beneficial to plants (Arrebola et al., 2019). Reports of plant beneficial rhizospheric *Pseudomonas* spp. producing different metabolites of interest for plants are common (Francis et al., 2010; Biessy and Filion, 2018), such as antifungals (Cazorla et al., 2006; Deng et al., 2015; Nandi et al., 2015), siderophores (Calderón et al., 2015), volatile compounds (Han et al., 2006; Nandi et al., 2015), insecticidal compounds (Flury et al., 2016; Arrebola et al., 2022) and some enzymatic activities (Flury et al., 2016; Raio et al., 2017).

To gain insight into the multitrophic interactions that could take place among the microbiota inhabiting the plant rhizosphere, the use of microbial synthetic communities (SynCom) as a model of reduced complexity to study plant microbial communities’ interactions, their relationship with the host, and interactions between their members has become a common bottom-up approach (Niu et al., 2017; Voges et al., 2019; Zhang et al., 2019). Moreover, the practical application of bacterial consortia in agriculture has been proposed as a potential way to improve crop production by enhancing synergic PGP activities and biocontrol abilities against phytopathogens. For this, antagonism against phytopathogens is a usual prerequisite for the selection of SynCom members (de Vrieze et al., 2018; Santhanam et al., 2019; Niu et al., 2020). Another important ability for plant beneficial consortia is the plant colonization. Traits such as bacterial motility and, more specifically, biofilm formation on plant surfaces, are crucial for efficient bacterial colonization of the plant (Pandit et al., 2020). Thus, combination of plant growth promoting strains that also have efficient plant colonization abilities, would be ideal for its use as consortia applications on globally important crops (Lally et al., 2017).

In previous studies, different *Pseudomonas* strains have been associated with healthy avocado (*Persea americana* Mill.) tree roots growing in an area affected by the soilborne phytopathogenic fungus *Rosellinia necatrix*, the causal agent of the avocado white root rot (Sztejnberg, 1980; Pliego et al., 2012). These *Pseudomonas* spp. were selected based on their broader antagonistic activity against *R. necatrix* and other soil-borne phytopathogenic fungi (Cazorla et al., 2006). Interestingly, the presence and isolation of *P. chlororaphis* strains in the avocado rhizosphere have been repeatedly observed in different avocado fields, displaying broad spectrum of antagonistic activities against phytopathogenic fungi and suggesting an important role of this bacterial species in beneficial interactions in the avocado rhizosphere.

In this study, we studied the bacterial interactions among three Pc strains isolated from the same habitat, the healthy avocado root, to initiate the future design and construction of a bacterial synthetic community as a potential model for the study of the multitrophic interactions taking place in the avocado rhizosphere. In this study, we analysed the competitiveness of three *P. chlororaphis* (*P. chlororaphis* PCL1606, *P. chlororaphis* PCL1601 and *P. chlororaphis subsp. piscium* PCL1607) that were previously isolated from different avocado rhizospheres displaying antagonistic activities against different phytopathogenic fungi (Cazorla et al., 2006; Calderón et al., 2014; Calderón et al., 2015). By a genome comparative analysis, bacterial potential traits that could be involved in the interaction with the plant were detected. To further explore the possible bacterial-plant interactions, the biofilm formation was studied as one of the main basis for bacterial plant colonization, and to visualize the *in vivo* root colonization by each selected bacterium, derivative bacterial strains expressing fluorescent proteins were constructed and visualized under confocal laser microscopy.

## 2 Materials and methods

### 2.1 Bacterial strains, plasmids, and growth conditions

The bacterial strains and plasmids used in this study are listed in **Table 1**. Each *Pseudomonas chlororaphis* strain used in this study, was isolated from roots of healthy avocado trees growing in avocado crop fields affected by *Rosellinia necatrix*, all located in the Málaga province (Spain; Cazorla et al., 2006). Routinely, the three wild-type *Pseudomonas chlororaphis* strains PCL1601, PCL1606 and PCL1607, and the derivative *Pseudomonas* spp. obtained were grown in lysogenic broth (LB) medium (Bertani, 1951), tryptone peptone glycerol (TPG) medium (Calderón et al., 2014), and M9 minimal medium supplemented with 10 mM succinate as a carbon source (Miller, 1972; Tienda et al., 2020). *Escherichia coli* was grown on LB. The rhizospheric biocontrol strain *Bacillus subtilis* PCL1608 (Bs08) was routinely grown on LB. Bacterial cultures were incubated at 25°C for 48 h (24 h) for *Pseudomonas* spp. and 37°C for 24 h for *E. coli* and *B. subtilis* strains. For solid plate media, 1.5% agar was supplemented. Media were supplemented with antibiotics, if necessary: kanamycin (km, 50 µg/ml, ampicillin (Amp, 50 µg/ml), gentamicin (Gm, 25 µg/ml), and rifampicin (Rif, 10 µg/ml).

**Table 1:** Microbial strains and plasmid used in this study.

Strain	Relevant characteristic	Reference
**Bacterial strains**		
***Pseudomonas chlororaphis***		
**PCL1601 (01)**	Wild type strain, isolated from avocado rhizosphere, producer of antifungal phenazines. Biocontrol against phytopathogenic fungi.	Vida et al., 2017
01-mCherry	Wild type 01 strain tagged with mCherry. Red fluorescence, Gm^r^	This study
**PCL1606 (06)**	Wild type strain, isolated from avocado rhizosphere, producer of antifungal 2-hexyl, 5-propyl resorcinol. Biocontrol against phytopathogenic fungi.	Cazorla et al., 2006
06-Rif^r^	Spontaneous mutant of 06, Rif^r^	González-Sánchez et al., 2010
06-Rif^r^-CFP	06-Rif^r^ tagged with eCFP. Cyan fluorescence, Rif^r^, Gm^r^	This study
***P. chlororaphis subsp. piscium***		
**PCL1607 (07)**	Wild type strain, isolated from avocado rhizosphere, producer of antifungal phenazines. Moderate biocontrol against phytopathogenic fungi.	Cazorla et al., 2006
07-GFP	Wild type 07 tagged with GFP. Green fluorescence, Km^r^	This study
***Bacillus subtilis***		
**PCL1608 (08)**	Wild type strain, isolated from avocado rhizosphere, producer of antifungal lipopeptides. Biocontrol against phytopathogenic fungi.	Cazorla et al., 2007
***Escherichia coli***		
S17-1 λpir mini-Tn*7*-*gfp-km*	Donor strain with a mini-Tn*7*-km harbouring gfp. Km^r^, Amp^r^	Norris et al., 2010
S17-1 λpir mini-Tn*7*-*mCherry-Gm*	Donor strain with a mini-Tn*7*-gm harbouring mCherry. Gm^r^, Amp^r^	Norris et al., 2010
S17-1 λpir mini-Tn*7*-*cfp-Gm*	Donor strain with a mini-Tn*7*-km harbouring cfp. Gm^r^, Amp^r^	Norris et al., 2010
S17-1 λpir pUX-BF13 (*tnsA-E*)	Helper strain with a 9.0-kbp *Eco*RI fragment containing *tnsABCDE*. Amp^r^	Bao et al., 1991
S17-1 λpir pTNS3	Helper strain for Tn*7* transposition. Amp^r^	Choi et al., 2006
**Fungal strains**		
***Rosellinia necatrix***		
CH53	Wild type strain, isolated from avocado root, cause avocado white root rot.	Pérez-Jiménez, 1997

The virulent fungal strain *R. necatrix* CH53 (López-Herrera and Zea-Bonilla, 2007; Pliego et al., 2008) was used for biocontrol experiments in the experimental pathosystem avocado/*R. necatrix*. The fungus was maintained by sclerotization (Gutiérrez-Barranquero et al., 2012) and routine growth on potato dextrose agar (PDA) plates at 25°C.

### 2.2 *In silico* analysis of genes and genomes

#### 2.2.1 Phylogenetic analysis

To perform a multiple sequence alignment and a phylogenetic study of the Pc strains used in this study, the available information from 43 genomes of rhizospheric *Pseudomonas* (**Supplementary File 1**) was used. A phylogenetic tree was constructed using MEGA 11 software (Biodesign Institute, Tempe, USA) by Multilocus Sequence Typing (MLST) using the concatenated housekeeping genes for *16S*, *rpoD*, *gyrB* and *recA* (Tamura et al., 2021; **Supplementary File 1**). The concatenated sequences were aligned using the Muscle algorithm (Edgar, 2004), the neighbour-joining method with 1000 bootstraps and the Juke-Cantor model. *Pseudomonas aeruginosa* PAO1 was used as an outgroup.

#### 2.2.2 Comparative genomics of Pc strains

The comparative genomics of the three rhizospheric Pc from avocado roots were analysed using the tools available in the EzBiocloud web page (www.ezbiocloud.net). Briefly, 20 *Pseudomonas* genomes, most of which were plant-related *Pseudomonas* spp. (**Supplementary Table 1**), were selected because of their previous use in similar studies (Biessy et al., 2019) and analysed. Using these web tools, all the genomes were annotated (including COG annotation), and the coding sequences (CDSs) were analysed by reciprocal best hits to determine the pan-genome orthologous groups (**Supplementary file 2**). Then, the comparison of the three Pc strains was extracted from the web-based tool, and unique genes, shared genes and core genes were listed in an Excel file (**Supplementary file 3**). For the percentage of COG calculations, all the CDSs of each group of genes were included, including those with no match in the database, termed NC (**Supplementary file 3**).

#### 2.2.3 Detection of PGP related traits

Genome analysis for PGP-related traits was performed by web-based Blastp (protein Basic Local Alignment Search Tool) in the Pseudomonas Genome database (www.pseudomonas.com) using the pre-established parameters (above 50% homology was considered a positive match). Gene sequences of all necessary proteins of the selected PGP activities were used to search the genomes of PCL1601, PCL1606 and PCL1607, except for very large biosynthetic gene clusters (BGCs), in which case the core protein genes of the clusters were used for searching. To corroborate the analysis, nucleotide nBlast (nucleotide Basic Local Alignment Search Tool; https://blast.ncbi.nlm.nih.gov; NCBI) was searched with the same list of gene nucleotides. Information for all the genes used for each PGP activity is listed in **Supplementary File 4**. For the detection of bacterial secretion systems, the web-based service EffectiveDB from the University of Vienna was used (https://effectors.csb.univie.ac.at/; Eichinger et al., 2016).

### 2.3 Construction of derivative *Pseudomonas chlororaphis* strains

Bacterial derivative strains were constructed to incorporate antibiotic resistance and the production of fluorescent proteins to allow for visualization under confocal laser scanning microscopy (**Table 1**). The previously obtained spontaneous rifampicin-resistant derivative 06 strain (González-Sánchez et al., 2010) was used in the experiments.

To obtain the fluorescent and antibiotic-resistant derivative *P. chlororaphis*, a triparental conjugation approach was performed, and strains were tagged using mini-Tn*7* system chromosomal integration (Lambertsen et al., 2004). To tag the bacterial strain PCL1607 with GFP (PCL1607-GFP, green fluorescence), *Escherichia coli* S17-1 λpir mini-Tn*7*-gfp-Gm (**Table 1**) was used as a donor and *E. coli* S17-1 λpir pTNS3 was used as a helper. *E. coli* S17-1 λpir mini-Tn*7*-mCherry-Gm and *E. coli* S17-1 λpir mini-Tn*7*-cfp-Gm (**Table 1**) were used as donors for mCherry (red fluorescence) and CFP (cyan fluorescence) to tag PCL1601 (PCL1601-mCherry) and PCL1606-Rif^r^ (PCL1606-Rif^r^-CFP), respectively, and *E. coli* S17-1 λpir pUX-BF13 (tnsA-E) was used as a helper (Bao et al., 1991). The protocol for triparental conjugation was as described in the supplementary information of Butaite et al. (2017). After that, the cells were plated in Pseudomonas Isolation Agar medium (PIA) supplemented with the corresponding antibiotics: gentamycin (Gm, 25 μg/ml); kanamycin (km, 50 μg/ml); and rifampicin (Rif, 10 μg/ml).

### 2.4 Antagonism analysis by plate assay

To check the compatibility of the three wild-type Pc strains, PCL1601, PCL1606 and PCL1607 (**Table 1**), the growth inhibition among each other was evaluated in agar plates with TPG, TPG 1:20 diluted, LB and LB 1:20 diluted. As an inhibition control, we used *Bacillus subtilis* PCL1608 (08), a bacterial strain also isolated from the avocado rhizosphere, which has been previously reported to be inhibited by PCL1606 (Cazorla et al., 2007). The strains were grown in liquid TPG overnight at 25°C and 150 rpm. Agar plates were surface-inoculated with the strain to test, and then all three *Pseudomonas* sp. (PCL1601, PCL1606 and PCL1607) and *Bacillus* sp. (PCL1608) were adjusted to an O.D. of 1 at 600 nm (approximately 10^9^ cfu/ml), and a 10 µl was drop inoculated on the plate surface. The plates were sealed and incubated in darkness at 25°C. After 2-3 days, the formation of an inhibition halo on the agar surface was checked. Four independent experiments with 5 replicates each one, were performed.

### 2.5 *In vitro* competitive index assay

To calculate the *in vitro* competitive index (CI), derivative-transformed Pc strains were tested on TPG-rich medium and M9 minimal medium supplemented with 10 mM succinate (M9+succ), following the procedure described by Macho et al. (2007) with slight modifications. Briefly, the three test strains (PCL1601, PCL1606 and PCL1607) were cultivated overnight with the corresponding antibiotic for further selection, washed twice with fresh liquid media and adjusted to 0.05 O.D. at 600 nm (approximately 10^5^ cfu/ml) for assay. Dual or triple (3P) combinations of the strains (1:1 and 1:1:1 proportions, respectively) were adjusted to a final volume of 5 ml into a tube. After 24 h of incubation, 10-fold dilutions were made and plated with the appropriate antibiotics for specific colony counting. The CI was calculated by determining the ratio of each strain to the total population. Three independent experiments, with 3 replicates each one, were performed.

### 2.6 Biofilm-related assays

#### 2.6.1 Colony morphology assay

As a model of controlled biofilm formation, a colony morphology assay was performed to determine the composition of the extracellular matrix, as described by Friedman and Kolter (2004), with modifications. Precultures of the three wild-type strains on TPG were adjusted to an O.D. of 0.8 at 600 nm (approximately 10^8^ cfu/ml), mixed in dual and triple combinations in equal proportions, and inoculated onto the plate surface. TPG plates supplemented with Congo red (40 µg/ml) and Coomassie brilliant blue (20 µg/ml) were drop-inoculated with 10 µl of single strains and dual, and triple combinations of the strains. The plates were then incubated at 25°C for 5 days to allow for colouring of the colonies, and images were captured using a stereomicroscope AZ-100 (Nikon Co., Tokyo, Japan). Five independent experiments with 5 replicates each one, were performed.

#### 2.6.2 Air-liquid pellicle formation assay

The *in vitro* formation of a pellicle at the air-liquid interface was visually analysed for the three single strains (PCL1601, PCL1606, and PCL1607) and their dual and triple combinations. Each strain was cultured on TPG at 25°C overnight (o/n), washed twice with fresh TPG media, adjusted to 0.8 O.D. at 600 nm (approximately 10^8^ cfu/ml) and mixed in equal proportions for each combination. Five microlitres of a single strain or dual or triple combination were inoculated in 1 ml of TPG placed into each well of a plastic 24-well plate. The plates were sealed and incubated in the dark at 25°C without movement. After 10 days of incubation, pellicle formation was visually analysed. Four independent experiments with 5 replicates each one, were performed.

#### 2.6.3 Adhesion quantification

To quantitatively evaluate the biofilm formation ability *in vitro*, the procedure described by O’Toole, 2011 was followed with slight modifications. The early biofilm formation (adhesion to the wall of the plastic wells) of the wild-type strains (PCL1601, PCL1606 and PCL1607) was analysed in liquid TPG and M9+succ media using 24-well plates after inoculating the individual Pc strains and their dual and triple combinations (1:1 for the dual and 1:1:1 for the triple combination). Briefly, bacterial liquid o/n cultures were washed twice in fresh media, adjusted to an O.D. of 0.8 at 600 nm (approximately 10^8^ cfu/ml) and mixed in the different combinations. Eighty microlitres of each combination was inoculated in 720 µl of fresh TPG media into an individual well. After 24 h of incubation in the dark at 25°C, the liquid media in each well was removed and carefully washed three times with water to eliminate all bacterial cells that did not adhere to the surface. Then, methanol was added to each well for 20 min at room temperature to fix the cells. The methanol was removed, and when the 24-well plates were completely dry, each well was stained with 1 ml of 0.1% crystal violet and incubated for 20 minutes at room temperature. The crystal violet was removed and carefully washed with tap water to remove any remaining dye. One millilitre of 33% acetic acid was added to each well and incubated for 20 minutes with soft agitation to solubilize all the stain adhered to the biofilm. Finally, the amount of solubilized crystal violet was measured using a spectrophotometer at 590 nm absorbance (O’Toole, 2011). Four independent experiments with 3 replicates each one, were performed.

#### 2.6.4 Visualization of submerged biofilm formation

A modified version of the biofilm assay described in 2.7.3. was performed using the fluorescent-labelled strains (PCL1601-mCherry, PCL1606-Rif^r^-CFP and PCL1607-GFP) to describe the bacterial distribution on an abiotic surface by confocal microscopy. For that, a cover glass piece of microscopy was introduced into 1 ml of fresh TPG placed into each well of a plastic 24-well plate to let bacteria adhere and form an early biofilm on its surface. After 24 h of incubation at 25°C, the cover glasses were removed and directly observed using confocal laser microscopy, and 2D and 3D images were obtained with Stellaris 8 equipment (Leica microsystems^©^). Three independent experiments with 5 replicates each one, were performed.

### 2.7 *In planta* assays

#### 2.7.1 Competitive index in avocado roots and visualization with confocal microscopy

The competitive index (CI) was also evaluated *in vivo* on avocado roots inoculated with the tagged-single strain inoculants (PCL1601-mCherry, PCL1606-Rif^r^-CFP and PCL1607-GFP), dual and triple combinations. Commercial 6-month-old avocado plants (cv. Walter Hole) were obtained from Brokaw nurseries (Brokaw España, S.L., Vélez-Málaga, Spain). The avocado plant roots were disinfected in 0.1% sodium hypochlorite solution (20 minutes), washed in sterile water for 20 minutes, and inoculated for another 20 minutes with bacterial cell suspension adjusted to an O.D. of 1.0 at 600 nm (approximately 10^9^ cfu/ml). The control plant roots were incubated with TPG liquid media as a reference. Inoculated plants were transferred to the appropriate substrate and placed into a greenhouse at 25°C for 24 h. For the CI assays, 1 g of roots was homogenized in 2 ml of saline solution (0.85%) for 3 minutes. Tenfold dilutions were plated on LB agar plates supplemented with antibiotics for proper counting. The CI was expressed as a percentage of every strain in the total population per gram of fresh avocado root. The assay was independently performed three times with five replicates each one.

From the same inoculated plants, superficial fresh root samples were taken after 24 h, placed on glass slides, and visualized under confocal laser scanning microscopy as previously described.

#### 2.7.2 Assay of bacterial persistence in avocado root assay

To determine bacterial persistence in avocado roots over time, 6-month-old commercial avocado plants (cv. Walter Hole) roots were disinfected, washed, and inoculated as previously described. Fifteen plants per treatment were used. The inoculated plants were maintained in a greenhouse and monitored for four weeks. Tagged strains (PCL1601-mCherry, PCL1606-CFP and PCL1607-GFP) were inoculated individually and with the three strains in combination (3P). Samples were taken at 1, 8, 14, 21 and 28 days postinoculation, and the assay was independently repeated three times. Three independent experiments with 4 replicates each one, were performed.

#### 2.7.3 Avocado-*Rosellinia necatrix* biological control assay

To determine the biocontrol ability of the three Pc strains (3P) combined and their action as a bacterial consortium, a biological control assay against the pathogen *Rosellinia necatrix* CH53 in avocado plants was performed as described previously (Cazorla et al., 2006). Briefly, roots of commercial 4-month-old avocado plants (cv. Walter Hole) were cleaned, disinfected and washed as previously described. For bacterial inoculation, roots were immersed for 20 minutes into a bacterial suspension of the combination of three strains previously adjusted individually to an O.D. of 1 at 600 nm (10^9^ cfu/ml). As a positive biocontrol treatment, inoculations were performed with the biocontrol bacterial strain PCL1606 (Cazorla et al., 2006). Once inoculated, the plants were placed in plastic pots with proper substrate. Fungal inoculation was performed using infected wheat grains as previously described (Cazorla et al., 2006). Fifteen plants were used for each treatment, and plants immersed in TPG medium were used as the control treatment. The plants were grown in a greenhouse at 25°C protected from direct sunlight. Plants were irrigated twice per week until the end of the experiment at 21 days. To determine the disease index (DI), aerial symptoms were annotated every 2-3 days during all experiments, and the DI was calculated as described previously (Cazorla et al., 2006). To quantify plant protection, the disease area under the disease progress curve was calculated in groups of 3 plants, and the data were statistically analysed (Arrebola and Cazorla, 2020). Three independent experiments with 5 replicates each one, were performed.

### 2.8 Statistical methods

All experiments were performed as at least three independent experiments. For statistical data analysis, the data distributions showed in **Figures 4E, F** (adhesion values) and 8 (biocontrol evaluated as the area under the disease progress curve), were tested using one-way analysis of variance (ANOVA) followed by Fisher’s least significant difference test with Bonferroni’s correction (*P* = 0.05). All data analyses were performed using IBM SPSS statistics 25 software (SPSS, Inc., Chicago, IL, United States).

## 3 Results

### 3.1 *In silico* comparisons of the Pc strains PCL1601, PCL1606 and PCL1607

**Figure 1:**
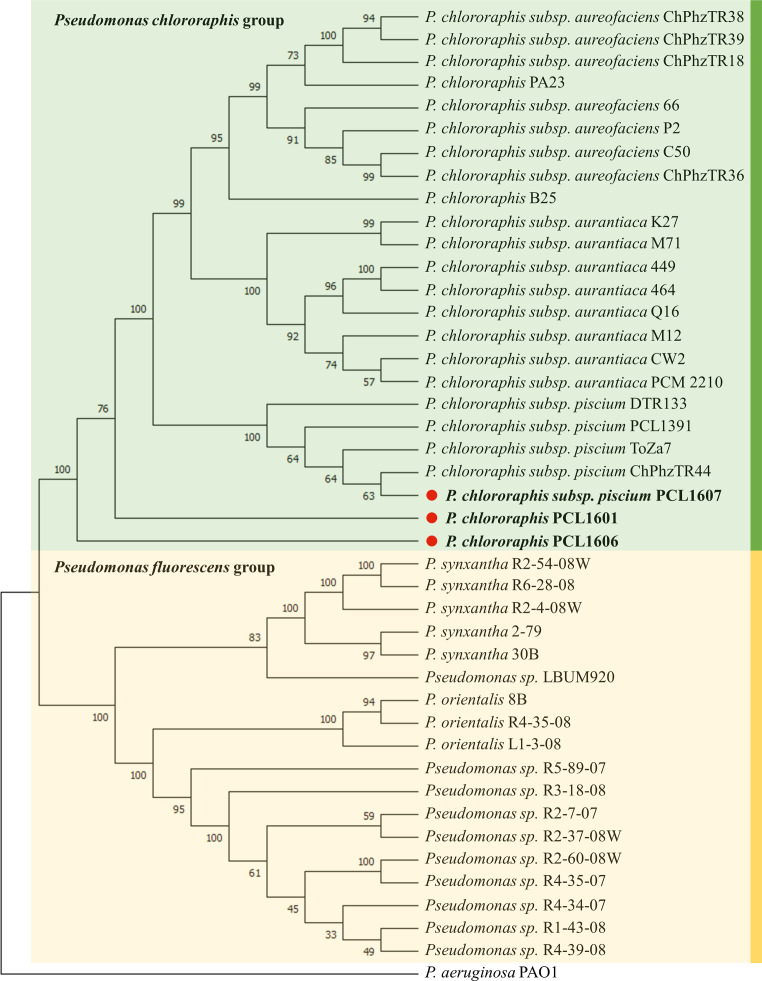
Rhizospheric *Pseudomonas* spp. phylogeny including Pc strains PCL1601, PCL1606 and PCL1607, marked with a red point and bold letters. Sequences were aligned by MUSCLE. The tree was made with MEGA 11 by multilocus sequence typing using concatenated 16S and the housekeeping genes *rpoD*, *gyrB* and *recA*. The neighbour-joining method with 1000 bootstraps and the Juke-Cantor model were used. The numbers in the nodes are the bootstrap values. *P. aeruginosa* PAO1 was used as an outgroup.

Phylogenetic analysis grouped the 43 *Pseudomonas* spp. in two main phylogroups, the *Pseudomonas fluorescens* group and *Pseudomonas chlororaphis* group (**Figure 1**). Interestingly, the rhizospheric strains PCL1606 and PCL1601 grouped with *P. chlororaphis*; however, these two strains form two different phylogenetic branches in the *P. chlororaphis* group apart from the rest of the *P. chlororaphis* strains included in this analysis. This study places strain PCL1607 into the branch comprised by other well-known biocontrol bacterial strains, such as ToZa7 and PCL1391.

The general features of the sequenced genomes of the three rhizospheric Pc strains are listed in **Supplementary Figure 2A**, showing similar features of size, G+C content, and number of coding sequences (CDSs). Only the strain PCL1607 displayed a higher number of unique CDSs when compared with 01 and 06.

Comparative analysis between the three Pc genomes is summarized in **Supplementary Figure 2B**, and the percentage of COG annotation in unique genes was calculated (**Supplementary Figure 2C**). Predicted proteins were functionally categorized using the COG database (Galperin et al., 2015), and the COG categories were compared among the genomes of PCL1601, PCL1606 and PCL1607 (**Supplementary Figure 2C**). Similar COG categories distributions among the three strains were observed. Interestingly, COG categories N (cell motility) and U (intracellular trafficking, secretion and vesicular transport), were absent in PCL1607. On the other hand, the percentage of COG categories C, L, M and V, representing energy production and conversion, replication, recombination and repair, cell wall membrane biogenesis and defence mechanisms, was higher in PCL1606. The results of these comparative genomic analyses are detailed in **Supplementary Files 2**, **3**, respectively.

**Figure 2:**
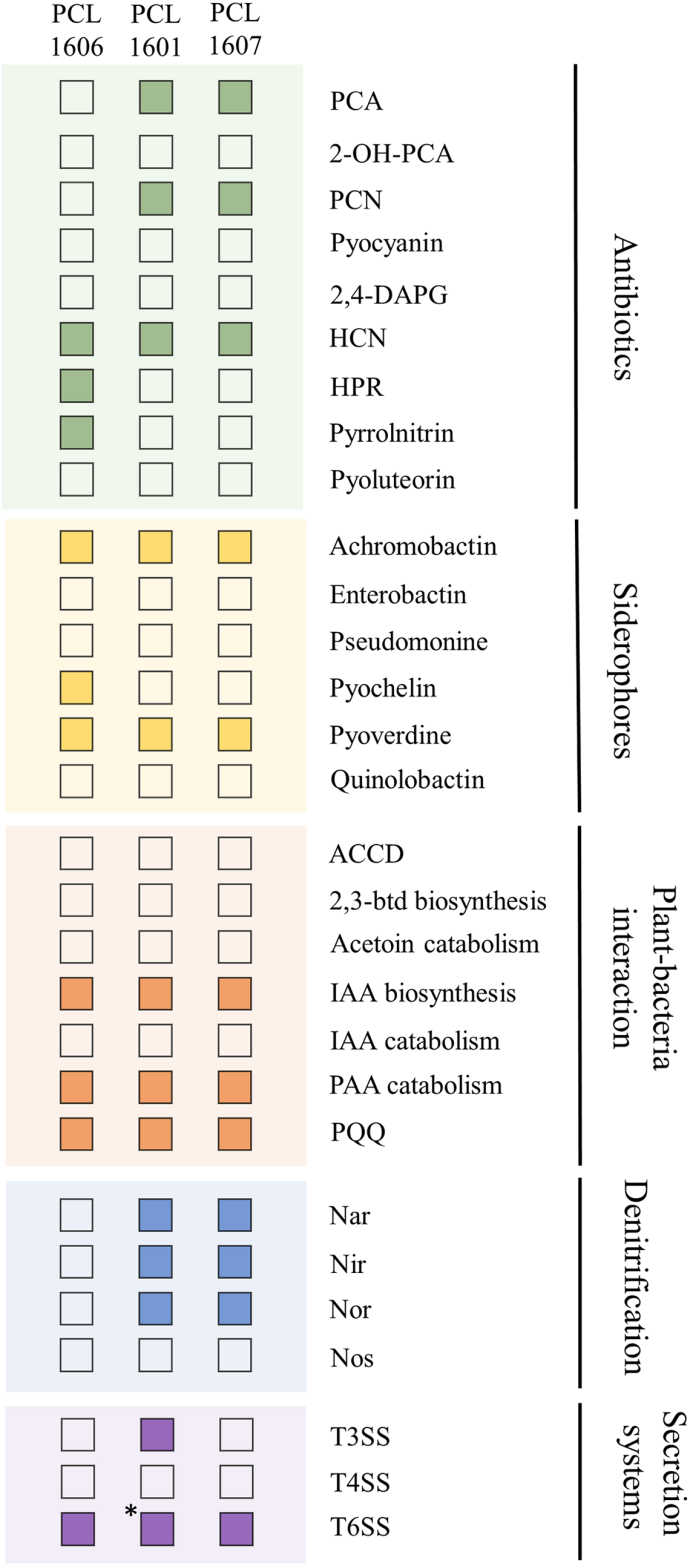
Positive (filled square) and negative (empty square) matches from a BLASTp search of PGP-related activities in the genomes of the selected Pc strains. PCA, phenazine-1-carboxylic acid; 2-OH-PCA, 2-hydroxyphenazine-1-carboxylic acid; PCN, phenazine-1-carboxamide; 2,4-diacetyphloroglucinol HCN, hydrogen cyanide; HPR, 2-hexyl-5-propyl resorcinol; ACCD, 1-aminocyclopropane-1-carboxylate deaminase; 2,3-btd, 2,3-butanediol; IAA, indol-3-acetic acid; PAA, Phenylacetic acid; PQQ, pyrroloquinoline. *: presence of 7 of 8 genes considered for a functional T3SS by software analysis.

Additionally, known PGP-related traits were searched *in silico* in the three Pc genomes (**Figure 2**). The three strains contain genes for the production of antifungal antibiotics (PCL1601 and PCL1607 harbour production genes of phenazines, and PCL1606 has genes for the production of 2-hexyl, 5-propyl resorcinol and pyrrolnitrin). For the production of siderophores, the three Pc strains have predicted genes for achromobactin and pyoverdine gene clusters, and PCL1606 also has genes with similarity to the pyochelin biosynthetic gene cluster. For the analysed plant-bacteria interaction activities, the three strains showed the same genetic features, but for denitrification activities, PCL1601 and PCL1607 have predicted genes related to the nar, nir and nor denitrification routes with no matched genes in PCL1606. Finally, the three strains have predicted genes for the type 6 secretion system (T6SS), but PCL1601 was the only strain showing the putative genes for a complete type 3 secretion system (T3SS).

### 3.2 Antagonism among test bacterial strains in the plate inhibition assay

The three rhizospheric Pc strains were tested for their compatibility by dual compatibility plate assays in LB, LB 1/20, TPG, TPG 1/20 media. In any combination, no clear inhibition halo was observed between the interacting Pc strains. However, a clear inhibition of the PCL1608 strain was observed for all the tested Pc strains, especially when using TPG medium. PCL1608 did not inhibit any Pc strain (**Supplementary Figure 1**).

### 3.3 Calculation of the competitive index in liquid media

**Figure 3:**
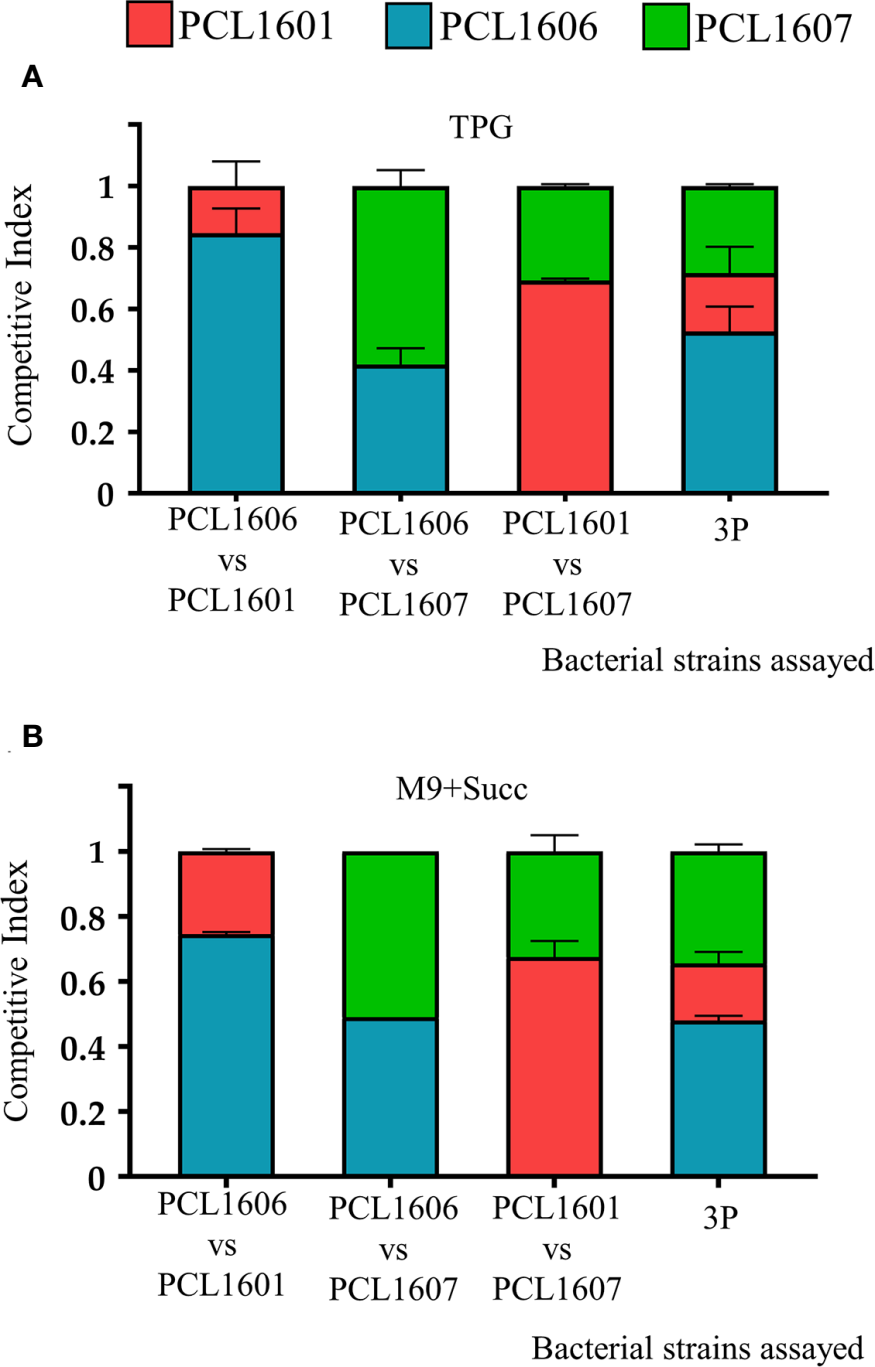
Competitive index (CI) in liquid media (TPG and M9+succ) calculated after 24 hours of growth at 25°C. All inocula were adjusted to an O.D. of 0.05 at 600 nm (10^5^ cfu/ml). Then, dual and triple inoculations were made at the same proportion of 1:1 (1:1:1) in **(A)** TPG and **(B)** M9+succ. *P. chlororaphis* PCL1601 (red); *P. chlororaphis* PCL1606 (blue); *P. chlororaphis subsp. piscium* PCL1607 (red); 3P: combination of three Pc strains. Average competitive index (bar) and standard error (error bars) of three independent experiments are presented.

To gain insight into the relationship between the three individual Pc strains, competitive index assays were performed with dual and triple combinations of the rhizospheric Pc strains using experiments on TPG and M9+succ liquid media. The results of the competitive indices (CIs) for the different combinations assayed were similar when using both TPG and M9+succ (**Figure 3**). In both rich and minimal media, PCL1606 overcame PCL1601 when they were combined, with CI values of 0.8 in TPG and 0.75 in M9+succ for strain PCL1606 in comparison to 0.2 in TPG and 0.25 in M9+succ for strain PCL1601. When PCL1606 and PCL1607 were combined, the CI was very similar but with a slight advantage of PCL1607 over PCL1606. The results for PCL1607 were 0.58 in TPG and 0.53 in M9+succ and those for PCL1606 were 0.42 in TPG and 0.49 in M9+succ. Finally, the combination of PCL1601 and PCL1607 showed higher values of PCL1601 over PCL1607, with a CI of 0.69 in TPG and 0.64 in M9+succ for PCL1601 in contrast to 0.31 in TPG and 0.36 in M9+succ for PCL1607. With all three *Pseudomonas* sp. (3P) in liquid media, the resulting CI showed that the PCL1606 strain was the dominant strain, with values of 0.53 in TPG and 0.49 in M9+succ, followed by PCL1607 with 0.28 in TPG and 0.36 in M9+succ, and finally by PCL1601 with 0.19 in TPG and 0.15 in M9+succ (**Figure 3**).

### 3.4 Analysis of the colony morphology

**Figure 4:**
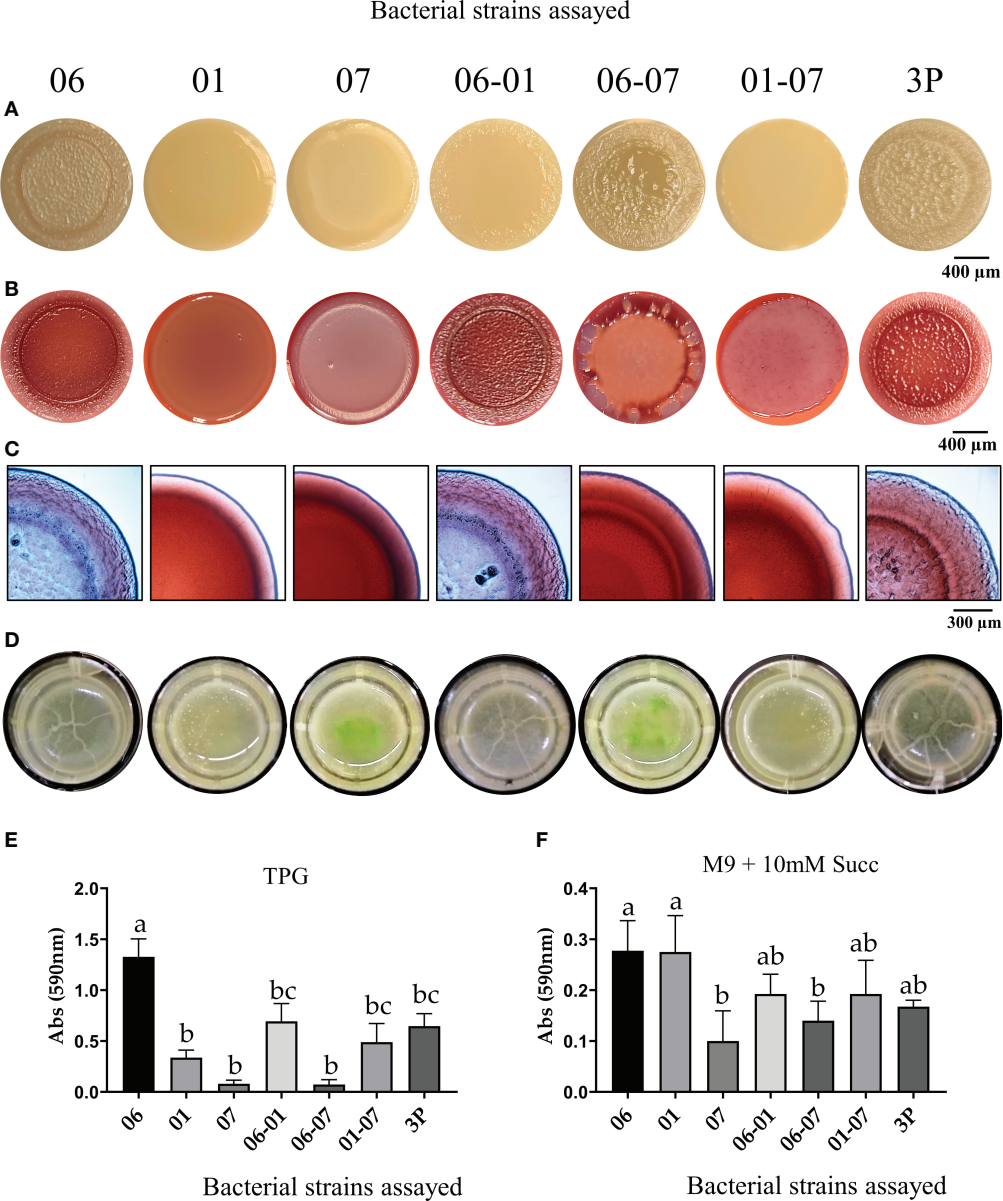
Colony morphology and biofilm formation of the individual strains and dual and triple strain combinations. Colony morphology growing after 5 days of incubation in **(A)** TPG, in **(B)** TPG plates supplemented with Congo red (40 µg/ml) and Coomassie brilliant blue (20 µg/ml). **(C)** Details of colonies grown on TPG supplemented with Congo red and Coomassie brilliant blue captured with a stereomicroscope. **(D)** Air-liquid interface pellicle formed after 5 days of incubation in TPG liquid media at 25°C. Single numbers correspond to single strain inoculation, and two numbers and 3P correspond to the dual and triple combinations of strains in the same proportion of 1:1 or 1:1:1, respectively. Average quantification and standard error of four independent experiments of bacterial adhesion are presented by measuring crystal violet absorbance at 590 nm in **(E)** TPG and **(F)** M9+succ. 01: *P. chlororaphis* PcPCL1601; 06: *P. chlororaphis* PCL1606; 07: *P. chlororaphis subsp. piscium* PCL1607; 3P: combination of the three Pc strains. Different letters represent a statistically significant difference after one-way analysis of variance, followed by Fisher’s least significance test (P = 0.05).

Bacterial colonies after 5 days of growth on agar plates of TPG media supplemented with Congo red and Coomassie blue showed that PCL1606 had the most complex colony morphology, presenting wrinkles and a heterogeneous surface with a dry and pale appearance, while PCL1601 and PCL1607 displayed a mostly homogeneous flat morphology with a wet and mucous-like appearance (**Figures 4A, B**). When the three Pc strains were combined, the principal phenotype of the interaction resembled to the dominant single-strain phenotype. In addition, the potential presence of exopolysaccharides (EPSs) is revealed by the ability to fix Congo red. Fixing Coomassie brilliant blue is more related to protein presence. The individual colonies of PCL1606, as well as the PCL1606-PCL1601 and 3P combinations, presented blue and red areas. However, the higher potential production of EPSs, revealed by an intense red colour staining, was present in all combinations containing PCL1607 (except 3P), and absent in those combinations containing PCL1606 (except the combination PCL1606-PCL1607). All the single and binary combinations contain displayed by PCL1601 alone (but not when combined; **Figure 4C**).

### 3.5 Characterization of biofilm-related traits

After 5 days of incubation in TPG liquid media, strain PCL1606, the combination PCL1606-PCL1601 and the triple combination (3P), showed a very similar thin layer pellicle with folds that cover the entire surface on the liquid surface. However, when testing single strains of PCL1601 and PCL1607, as well as the combinations PCL1606-PCL1607 and PCL1601-PCL1607, they showed a homogeneous and cloudy culture with green precipitation in PCL1607 and PCL1606-PCL1607 cultures (**Figure 4D**).

To quantify the early biofilm formation (or adhesion), the crystal violet quantification method (O’Toole, 2011) was performed in TPG and M9+succ media (**Figures 4E, F**). The results obtained with TPG medium showed that single inoculation with PCL1606 had the highest absorbance and strain PCL1607 the lowest absorbance. When the strains were combined in pairs, the PCL1606-PCL1601 combination showed the higher values, and PCL1601-PCL1607 combination the lowest. Finally, the mixture of all three strains resulted in Abs values of 0.65, similar to the values displayed by the combination PCL1606-PCL1601 (**Figure 4E**). On the other hand, the results obtained in the M9+succ minimal medium showed more reduced absorbance values for all strains, and less differences were observed among inoculated strains were observed (**Figure 4F**).

Additionally, the early biofilm formation on a glass cover slip surface under confocal laser scanning microscopy was visualized (**Supplementary Figure 3**). The single inoculations revealed that PCL1607 fails to completely cover the glass cover slip surface. The combinations of two strains (**Supplementary Figures 3D-3F**) showed a biofilm mostly composed by a dominant strain in each case. Finally, when all three *Pseudomonas* sp. were combined (**Supplementary Figure 3G**), the biofilm was composed of a higher proportion of PCL1606 with lower levels of PCL1607 and PCL1601 cells.

### 3.6 Avocado root-related traits

To unravel the interaction phenotype of the three Pc bacterial strains, and their use as a potential consortia, the following studies were performed on the avocado roots.

#### 3.6.1 Competitive index in avocado roots

**Figure 5:**
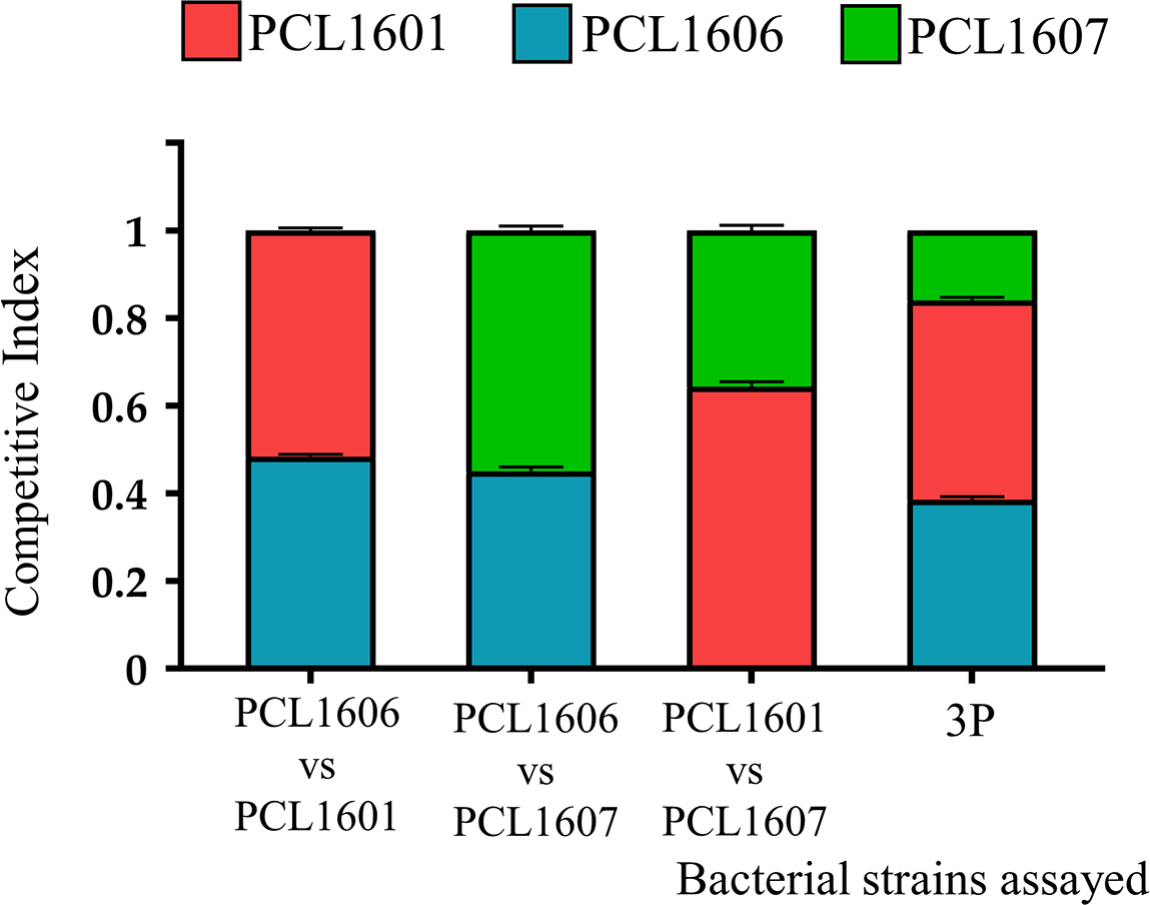
Competitiveness of Pc strains in avocado plant root assays. The competitive index in plant roots was calculated after 24 h of incubation. *P. chlororaphis* PCL1601 (red); *P. chlororaphis* PCL1606 (blue); *P. chlororaphis subsp. piscium* PCL1607 (red); 3P: combination of three Pc strains. Average competitive index (bar) and standard error (error bars) of three independent experiments are presented.

The analysis of the competitive index after 24 h in avocado roots (the natural environment where the test Pc were originally isolated), revealed that PCL1606 and PCL1601 showed not to be affected by any of the other Pc strains; however, PCL1607 seems to be challenged in presence of PCL1601 (**Figure 5**). Thus, the combination of PCL1606-PCL1601 in roots showed similar CIs (0.48 and 0.52, respectively), and both strains were in similar proportions in roots after 24 hours. When PCL1606 and PCL1607 were combined, the CI in avocado roots was also similar, 0.45 for PCL1606 and 0.55 for PCL1607. Finally, the combination of all three *Pseudomonas* sp. resulted in similar CIs for PCL1601 (0.45) and PCL1606 (0.39) and lower CI values (0.16) corresponding to PCL1607 (**Figure 5**).

#### 3.6.2 Persistence in avocado roots and colonization pattern

**Figure 6:**
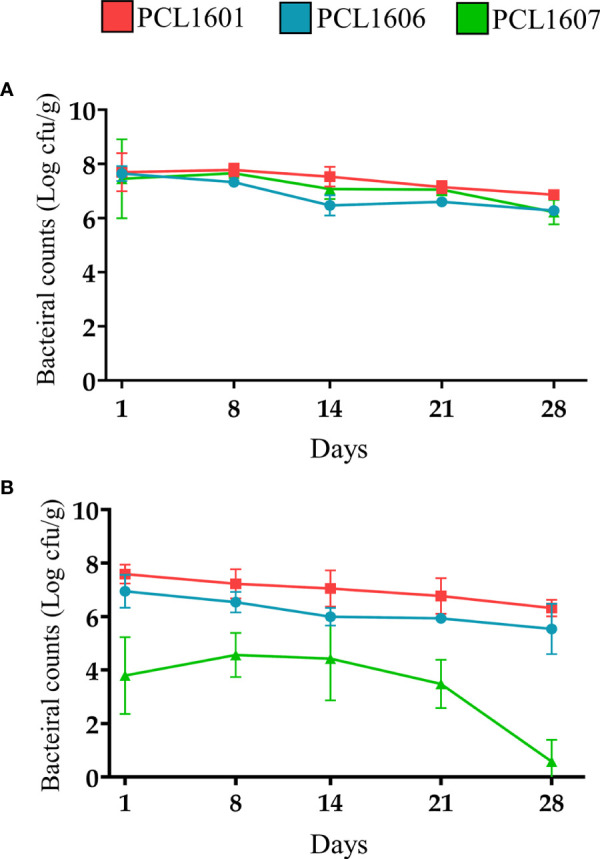
Time course of the bacterial counts of Pc strains inoculated in avocado roots **(A)** individually and **(B)** in triple combination (3P). *P. chlororaphis* PCL1601 (red); *P. chlororaphis* PCL1606 (blue); *P. chlororaphis subsp. piscium* PCL1607 (green). Bacterial counts and standard error (error bars) of three independent experiments are presented.

Experiments to establish the persistence of all three *Pseudomonas* sp. (PCL1601, PCL1606 and PCL1607) in avocado roots were performed by monitoring the bacterial counts on roots over 28 days. For that, single strains and the 3P combination were inoculated (**Figure 6**). Bacterial counts for inoculations of the single strains PCL1601, PCL1606 and PCL1607 resulted in similar values throughout the 28 days of the assay, displaying stable values above 10^6^ cfu/g of fresh root at the end of the assay (**Figure 6A**). However, when the avocado roots were inoculated with the three strains together, only PCL1601 and PCL1606 maintained a similar behaviour to the observed bacterial counts after individual inoculation on the root, while the counts of PCL1607 decreased by several orders of magnitude in the first day and almost no bacterial count was detected at the end of the assay (**Figure 6B**).

**Figure 7:**
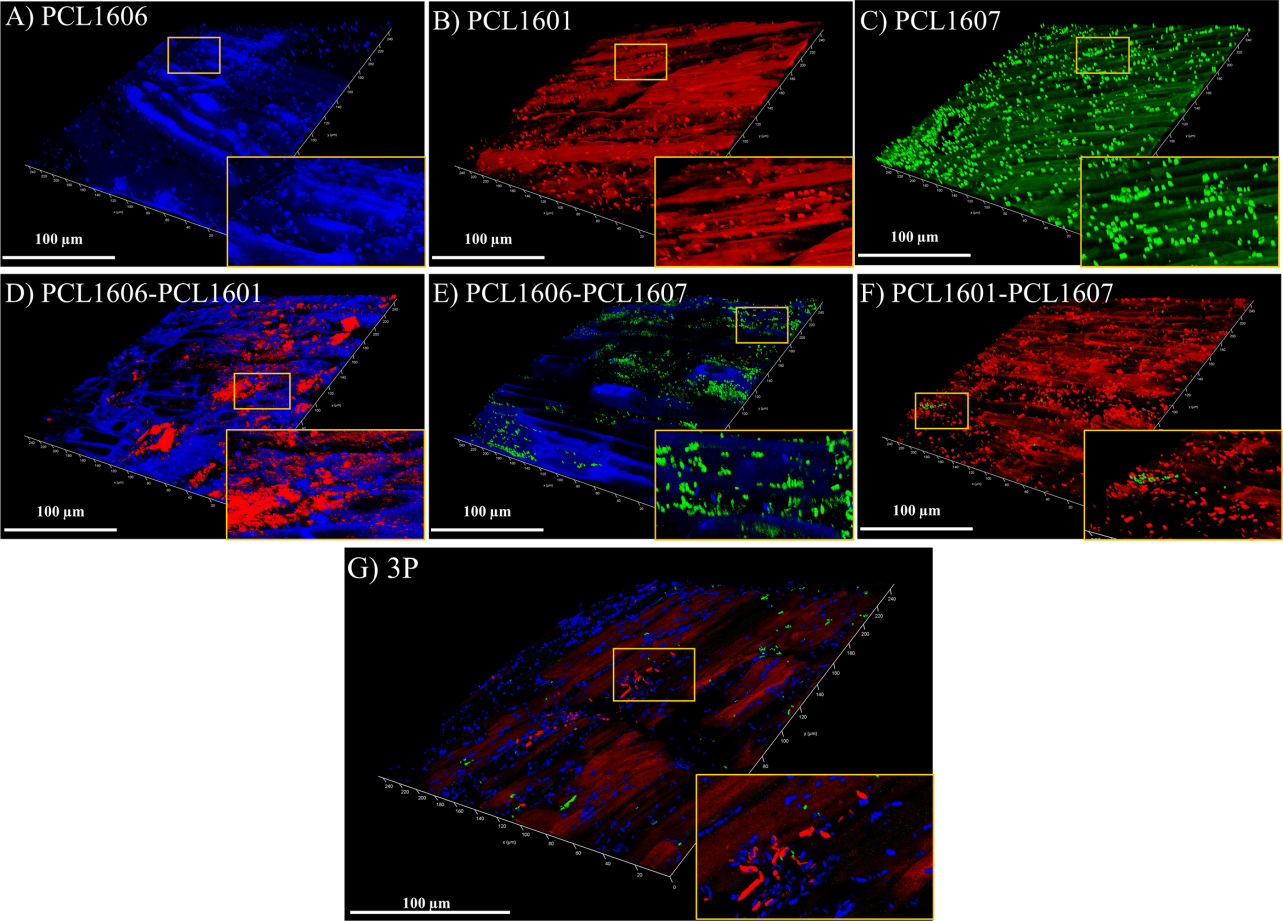
Bacterial distribution pattern on avocado roots after 24 h visualized under confocal laser microscopy. Single-strain biofilms **(A–C)** are shown: *P. chlororaphis* PCL1606 (in blue); *P. chlororaphis* PCL1601 (in red); and *P. chlororaphis subsp. piscium* PCL1607 (in green), as well as the dual and 3P combinations **(D–G)**. Yellow squares shown in the bottom right corner images correspond to areas magnified three times. Scale bars represent 100 µm. 3P: Three-strain combination.

To visualize the root colonization pattern in single strains and dual and triple combinations of the Pc strains, tagged strains were visualized using confocal microscopy (**Figure 7**) at 24 h post inoculation. The inoculated roots showed microcolonies when PCL1606 or PCL1601 colonized the avocado roots, but PCL1607 showed a dispersed bacterial distribution on the root. When the strains were combined in pairs, PCL1606 predominated over PCL1601. Combinations of PCL1606 and PCL1607 shared and occupied the same space. Finally, when inoculated with the combination of PCL1601- PCL1607, a predominance of PCL1601 was observed. The combination of all three *Pseudomonas* sp. showed a predominance of PCL1606, followed by PCL1601, with PCL1607 being less represented (**Figure 7**; **Supplementary Figure 4**).

#### 3.6.3 Biological control assay

**Figure 8:**
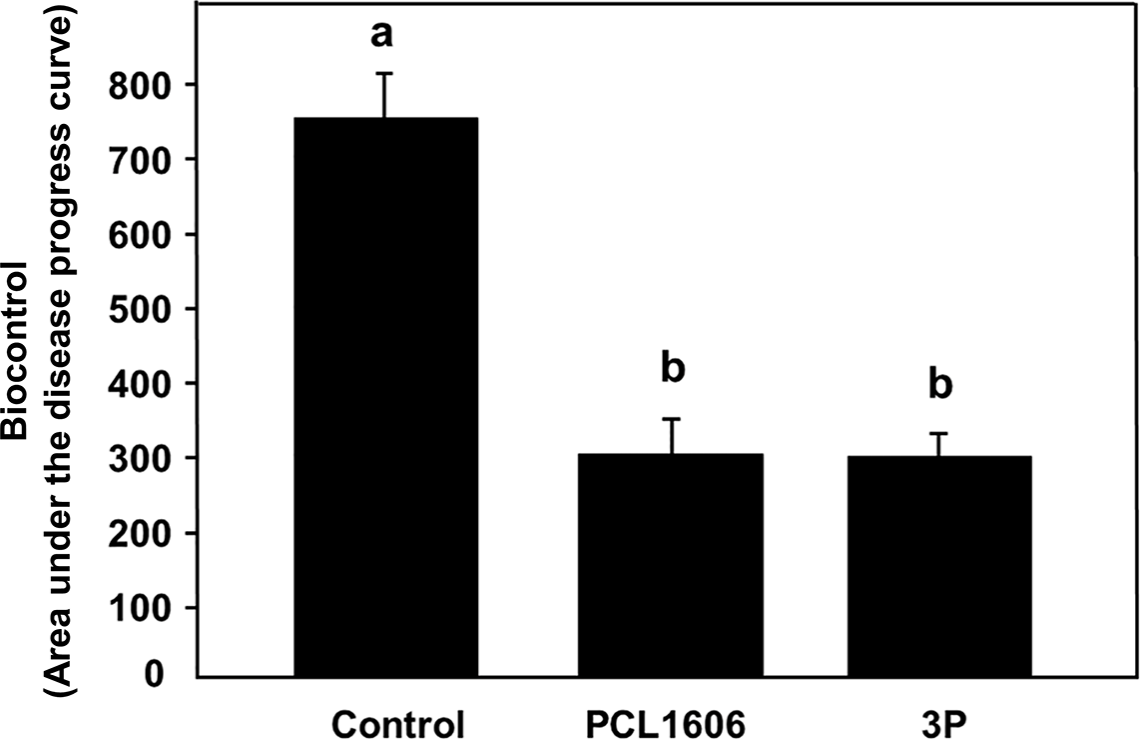
Biocontrol ability of combined 3 rhizospheric Pc strains (3P) against white root rot of avocado plants caused by *Rosellinia necatrix. P. chlororaphis* PCL1606 was used as a positive biocontrol reference. The area under the disease progress curve was calculated and analysed for significance after arcsine square root transformation with analysis of variance, followed by Fisher’s least significant difference test (P = 0.05). Average AUDPC values and standard error (error bars) of three independent experiments are presented. Values with different letters represent a statistically significant difference. 3P: Three-strain combination (*P. chlororaphis* PCL1601, *P. chlororaphis* PCL1606 and *P. chlororaphis subsp. piscium* PCL1607).

Using the avocado-*R. necatrix* test system, the disease index (calculated based in the aerial symptoms of avocado white root rot) have been monitored for each treatment during 21. The analysis of the resulting graph, allowed calculation of the area under the disease progress curve (AUDPC). AUDPC values obtained indicates that the treatment with 3P combined showed a protective phenotype against the fungal phytopathogenic fungi *R. necatrix*, with the same protection level as the positive control in plant protection, the wild-type *P. chlororaphis* 06 strain (showing average values of 297.1 ± 48.6 and 298.2 ± 52.3, respectively). These AUDPC values are statistically lower than that of the negative untreated control, which its higher AUDPC values (748.8 ± 59.9; **Figure 8**) demonstrates a faster appearance and higher severity of the avocado white root rot symptoms development.

## 4 Discussion

Currently, it is accepted that the observation of different microbial events that occur during plant interactions cannot be explained by the interaction of just one microorganism (Palková, 2004; Berg et al., 2020; O’Banion et al., 2020). Additionally, different genotypes of the same bacterial species can join the same plant habitat (Fromin et al., 2001), and at the same time, plants can influence the microbial composition of the microbial communities interacting with them (Rheault et al., 2020). One way to gain insight into the multitrophic interactions taking place in plant habitats is the use of synthetic communities (SynCom), which are considered a useful approach to manipulate and better understand groups of microorganisms acting in natural communities (Liu et al., 2022). Thus, the use of a SynCom approach could help to understand the rhizospheric colonization and persistence mechanisms observed for different representatives of *Pseudomonas chlororaphis* (Pc) that have been consistently isolated from avocado roots and also have an important role in pathogen suppression (Cazorla et al., 2006; González-Sánchez et al., 2010).

The three Pc strains selected in this study were previously isolated from different avocado rhizospheres in a search program for potential biocontrol microbial agents against soilborne fungal pathogens. These strains were selected because of their isolation from avocado roots and beneficial traits against fungal phytopathogens, although PCL1607 only displayed moderate plant protection activity (Cazorla et al., 2006). These strains, classified as Pc, showed different phenotypes and genotypes, in agreement with the results obtained by Biessy et al. (2019). Interestingly, Pc strains PCL1601 and PCL1606 are placed in two different phylogenetic branches apart from the rest of the Pc strains included in this analysis, confirming their phylogenetic differences among the selected Pc strains. On the other hand, bacterial strain PCL1607 grouped together with other beneficial *P. chlororaphis* subsp. *piscium* strains previously described to be associated with herbaceous plants, such as the strains ToZa7 (Kamou et al., 2015) and PCL391 (Chin-A-Woeng et al., 1998), suggesting a potential preference for a nonwoody root habitat. These differences were further confirmed after genome analysis, where the unique predicted CDSs could provide each strain with “individual” putative properties to help it in its natural habitat. The COG protein category analysis of the unique groups of predicted CDSs of each strain revealed that the percentage of genes with COG C, L, M and V annotations, representing energy production and conversion, replication, recombination and repair, cell wall membrane biogenesis and defence mechanisms, was higher in 06 than in the other two strains, indicating that this strain would be more suitable for survival in the root environment and may promote its adaptability to the changes of the habitat better than PCL1601 and PCL1607. Initially, the three *Pseudomonas* spp. strains were compatible with each other under lab conditions, since they did not show inhibition halos in any of the media assayed in the inhibition plate assay. Thus, the potential production of bacteriocins, such as R-Tailocins 1 and 2, which have been previously detected in strain PCL1606 with a predicted role in competition against other Pseudomonas (Dorosky et al., 2017), was not observed with the selected strains under the experimental conditions. Taken together, these results indicate that these three Pc strains may be compatible, which is a very important point to take into account when constructing a bacterial SynCom, especially when they are of the same species (Niu et al., 2020; Palmer and Foster, 2022).

When the interaction among strains was further investigated by calculating the competitive index (CI) on rich and minimal culture media, strain PCL1606 efficiently outcompeted the other Pc strains (in double or triple combinations). The resulting interactions among the different Pc selected were not dependent on the composition of the tested media, but an increased CI was consistently observed for PCL1606 when using the rich media, revealing a possible copiotrophic behaviour that has been described for some microbes of soil environments with high organic matter input (Koch, 2001; Lladó and Baldrian, 2017) and supports its high adaptability to soil and root environments after the *in silico* analysis. This is in agreement with previous observations that beneficial Pc strain presence was enhanced in avocado soils after organic amendments with composted almond shells (Vida et al., 2016).

Interestingly, the CI results obtained on culture media were not equivalent to those obtained from the avocado root, suggesting that results from culture media, even when trying to mimic the natural conditions of the root (such as using M9+succ medium), cannot be extrapolated to the natural habitat, as has also been previously reported (Palková, 2004; Tovi et al., 2021). Soil and rhizospheric environments are specific habitats where bacterial interactions and competition take place (Bai et al., 2015). Plant root exudates contain many primary metabolites, such as carbohydrates, amino acids, and organic acids, which bacteria can use to take advantage of over other competitors, depending on their metabolic capacity (Sasse et al., 2018). Additionally, root exudates can determine the composition of the rhizomicrobiome and can modify competitive interactions among bacteria (Mavrodi et al., 2021). In this study, the modifications of the bacteria-bacteria interactions induced by the avocado root were observed among strains, and no synergic effects were observed, indicating that competition for root tissue is the main interaction that drives root microbial community establishment, as it has been proposed in other experimental systems (de Kroon et al., 2003). Taken together, the phylogenetic results and differential COG categories (cell motility, intracellular trafficking, secretion and vesicular transport, signal transduction mechanisms, and defence mechanisms) suggest that PCL1607 would be less prepared for competition in the avocado root habitat, which was confirmed by root experiments on bacterial competition.

To unravel the behaviour of the Pc strains on the root scenario, the distribution pattern of fluorescent-tagged strains over the root surface viewed by confocal microscopy revealed a similar distribution pattern for the three strains when inoculated individually, colonizing the junctions of the epidemic cells of the roots, similar to that previously shown for the 06 strain (Calderón et al., 2014). However, when competing with other strains, PCL1607 did not efficiently compete when PCL1601 was present, displaying a cell distribution in dispersed microcolonies along the root surface and alteration of its previously described individual colonization pattern which was stable over time, approaching levels of approximately 10^6^ cfu/g root, which have been considered the carrying capacity for bacteria of the avocado root (Tienda et al., 2020). These results revealed that, individually, these strains can easily adapt to the root environment without competition with another microorganism. However, the cell distribution along the root surface changed when co-inoculations were performed, especially with 3P inoculation. With 3P, both the PCL1601 and PCL1606 strains showed a phenotype similar to the individual inoculations, confirming their compatibility with the root environment, which was interestingly not observed on media culture. Strain PCL1607 was observed to have the worst potential to compete and survive on avocado roots during competition. In contrast, more than 6 months of avocado root persistence and survival have been described for strain PCL1606 (Tienda et al., 2020). Independently, the biological control of avocado white root rot by the bacterial consortium did not show differences from the biocontrol strain PCL1606 alone, displaying the same level of plant protection. This result confirms the absence of synergies among the components of the bacterial consortium, and the strains that can persist on avocado roots have a protective phenotype on the roots.

The changes in the cell distribution pattern on the avocado root surface suggest a failure to colonize the roots of some Pc strains when others were present in the habitat. In the root environment, the presence of competitor strains modifies the rhizospheric lifestyle of surrounding microbes to result in a stable community, with some less-adapted members that can be excluded progressively. Since biofilms can be considered a key step in colonization (Noirot-Gros et al., 2018; Calderón et al., 2019; Zboralski and Filion, 2020), we revealed the role of biofilms in the bacterial consortium and bacteria-bacteria interaction phenotypes. The extracellular matrix is related to biofilm formation, and its composition determines biofilm characteristics (Mann and Wozniak, 2012; Fong and Yildiz, 2015). A first approach revealed differences in the dye binding of bacterial colonies growing on media culture plates, indicating differential extracellular matrix components that confirm the differences in the selected Pc strains (Solano et al., 2002; Sabnis, 2010). The investigation of pellicle and biofilm formation *in vitro* over an immersed microscope glass cover revealed that strain PCL1607 showed a heterogeneous pattern, not correlated with the lower staining by Congo Red dye. In the liquid-air biofilm formation analysis, only those combinations harbouring PCL1606 showed pellicle formation and failed in staining with Congo red, with the exception of combination PCL1606-PCL1607, suggesting a minor role of exopolysaccharides in the biofilm formation by these bacteria under the assayed conditions. On the other hand, the combinations containing PCL1607 produced green colouring of the media, as has been previously observed for other *P. chlororaphis* sp. *piscium* strains associated with the production of phenazine-1-carboxamide (Chin-A-Woeng et al., 1998). The combination of PCL1601-PCL1607 did not produced greenish colour of the media, suggesting that PCL1601 can inhibit the production of these compounds, thus altering the phenotype of PCL1607, and this change could be involved in the recovery of pellicle formation in the 3P assays. Bacterial motility lifestyle and sessile lifestyle (biofilm formation) are oppositely regulated, and these changes in lifestyle are primordial for adaptation and colonization of plant roots (Kolter and Greenberg, 2006; Zboralski and Filion, 2020b). Changes in environmental metabolites are the main guide for this switching in lifestyle (Maddula et al., 2006), and the modification of the components of the medium might be the reason for this change in biofilm formation ability.

Our research suggests that studies dealing with microbial interactions in natural habitats, can be benefited by *in vitro* studies to unravel the molecular bases of crucial microbial traits. However, the final microbial interaction *in vivo*, on their natural habitat and under the natural environmental conditions, must be tested to uncover the real role of such microbial traits. The studies using microbial consortia, and the assemblage of SynComs, are a recent approach that tries to resemble the natural situation of microbial communities, increasing the number of microbial actors in the experiments, but at the same time reducing the complexity find in nature that could allow its study. To our opinion, complexity of the future SynComs can be increased using current ‘omics techniques that provide knowledge of the microbial communities interacting in the natural habitats, their activities and their influence on this habitat, and allowing their study in the natural environment.

## 5 Conclusion

With these results, we confirm that a bacterial consortium composed of the beneficial Pc strains 01, 06, and 07 can have plant protection abilities, highlighting that 2 out 3 P*. chlororaphis* strains (PCL1601 and PCL1606) within this consortium are fully compatible, as they efficiently colonize avocado roots. The third strain (PCL1607) displayed reduced competitiveness under the conditions assayed in this study, probably due to difficulties forming biofilm, less competitive ability, or the possibility of adaptation to a different environment more related to herbaceous plants, then resulting in exclusion from the avocado root habitat. These results suggest that strain PCL1607 could be a candidate for substitution in a future bacterial consortium, and new avocado root-associated strains with better fitness in the presence of PCL1601 and PCL1606 could be selected and explored in further studies to improve a more complex SynCom that will have an important future role in sustainable plant growth.

## Data availability statement

The original contributions presented in the study are included in the article/**Supplementary material**. Further inquiries can be directed to the corresponding author.

## Author contributions

RV-M, EA and FC designed the experiments. RV-M, ST, and JG-B performed the experiments. RV-M, AV, VC, EA and FC analyzed the results and wrote the manuscript. All authors contributed to the article and approved the submitted version.

## Supplementary material

Supplementary Figure 1 Antagonism among rhizobacteria tested by plate assay in different growth media (LB, TPG, and both media diluted 1/20). **(A)** Scheme of the bacterial inoculation plates. **(B)** Antagonism plate assay results. PCL1601: *P. chlororaphis* PCL1601; PCL1606: *P. chlororaphis* PCL1606; PCL1607: *P. chlororaphis subsp. piscium* PCL1607; PCL1608: *B. subtilis* PCL1608.

Supplementary Figure 2 Comparative genomics of Pc strains PCL1601, PCL1606 and PCL1607. **(A)** General genome features of the three Pc strains. **(B)** Venn diagram representing the comparative genomics. Numbers indicate the CDS counts; **(C)** Percentage of COG categories in the unique CDS of Pc strains PCL1606, PCL1601 and PCL1607 calculated over the total CDS of each group. NC: no categorization in any COG category.

Supplementary Figure 3 Early biofilm formation on a cover glass slip surface after 24 h of immersion in inoculated TPG medium visualized by 3D confocal laser scanning microscopy. Single-strain biofilms **(A–C)** are shown: *P. chlororaphis* PCL1606 (in blue); *P. chlororaphis* PCL1601 (in red); and *P. chlororaphis subsp. piscium* PCL1607 (in green), as well as the dual and 3P combinations **(D–G)**. Biofilm details are shown in yellow squares magnified 3 times. The scale bars represent 100 µm. 3P: Three-strain combination.

Supplementary Figure 4 2D visualization under confocal laser microscopy of the bacterial population established on avocado roots after 24 hours. Pictures correspond to inoculated single, dual and triple combinations of *P. chlororaphis* strains. *P. chlororaphis* PCL1601 (red), *P. chlororaphis* PCL1606 (blue) and *P. chlororaphis subsp. piscium* PCL1607 (green). 3P: Three *Pseudomonas* sp. strains combined. Scale bars represent 80 µm.
