# Supplementary figures and images for: Interplay between rhizospheric Pseudomonas chlororaphis strains lays the basis for beneficial bacterial consortia
Source: Front Plant Sci. 2022 Dec 15;13:1063182. doi: 10.3389/fpls.2022.1063182 (PMC9797978; doi:10.3389/fpls.2022.1063182)

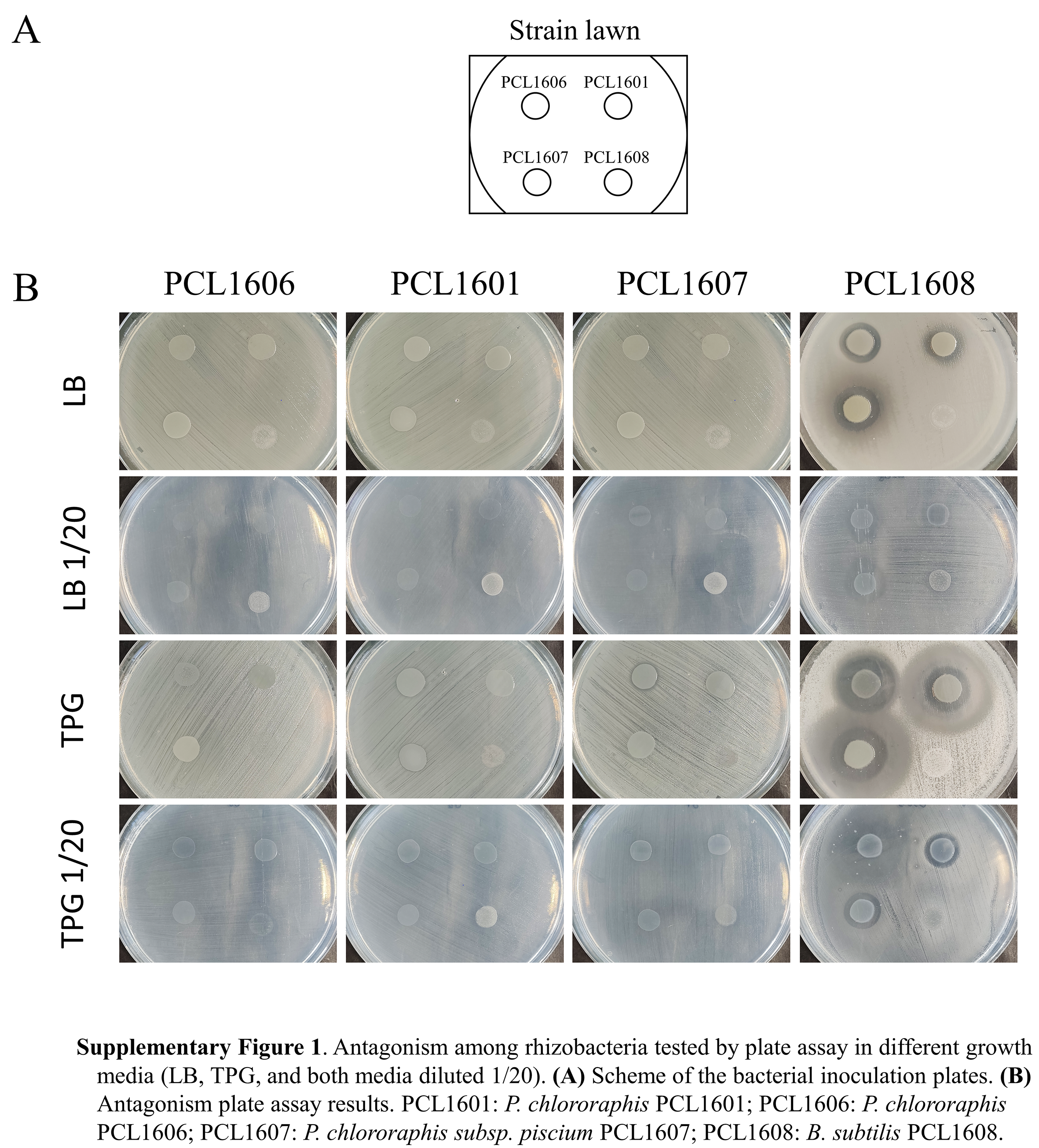

Supplement: Supplementary Figure 1 — Antagonism among rhizobacteria tested by plate assay in different growth media (LB, TPG, and both media diluted 1/20). (A) Scheme of the bacterial inoculation plates. (B) Antagonism plate assay results. PCL1601: P. chlororaphis PCL1601; PCL1606: P. chlororaphis PCL1606; PCL1607: P. chlororaphis subsp. piscium PCL1607; PCL1608: B. subtilis PCL1608. [file Image_1.tif]

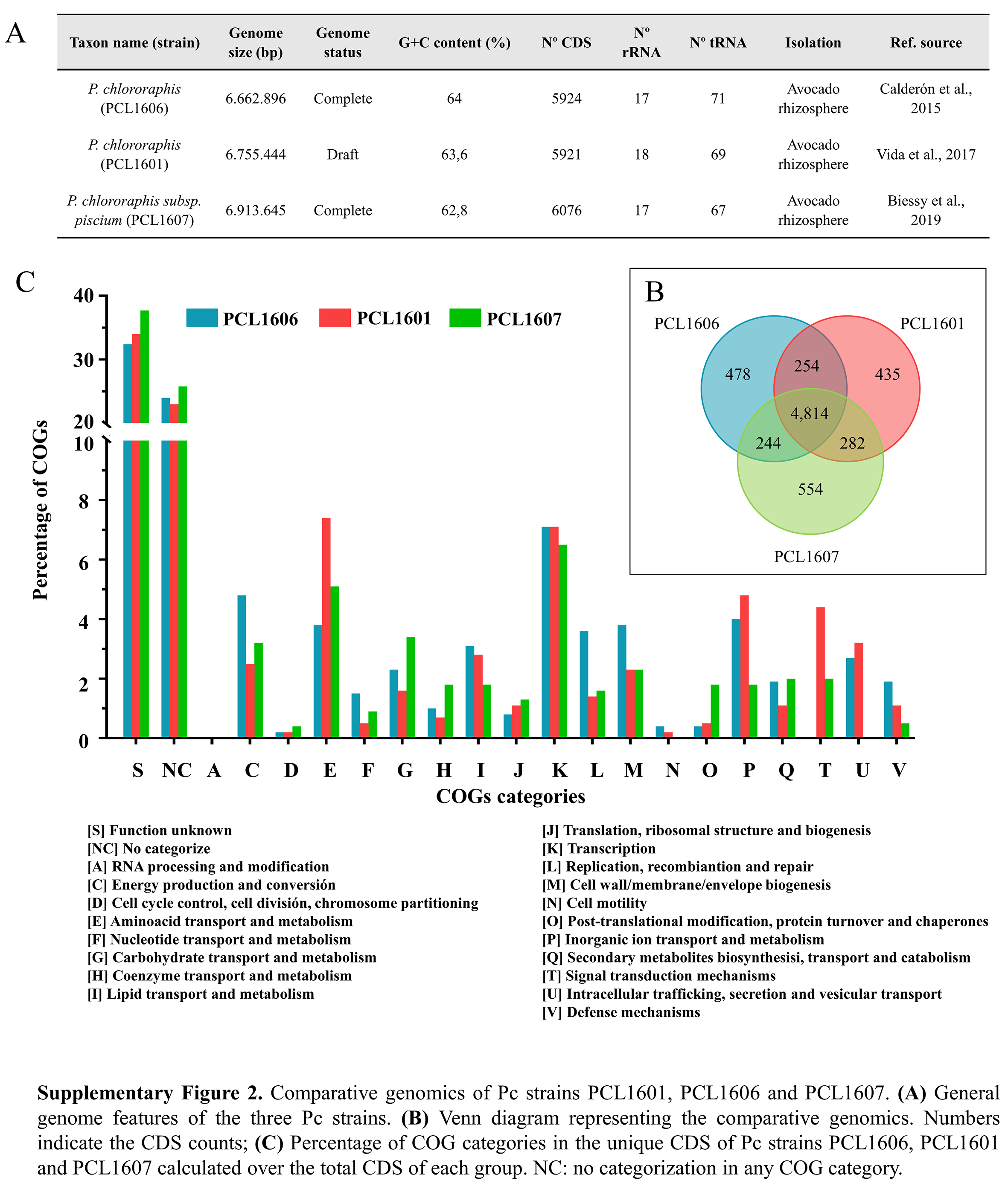

Supplement: Supplementary Figure 2 — Comparative genomics of Pc strains PCL1601, PCL1606 and PCL1607. (A) General genome features of the three Pc strains. (B) Venn diagram representing the comparative genomics. Numbers indicate the CDS counts; (C) Percentage of COG categories in the unique CDS of Pc strains PCL1606, PCL1601 and PCL1607 calculated over the total CDS of each group. NC: no categorization in any COG category. [file Image_2.tif]

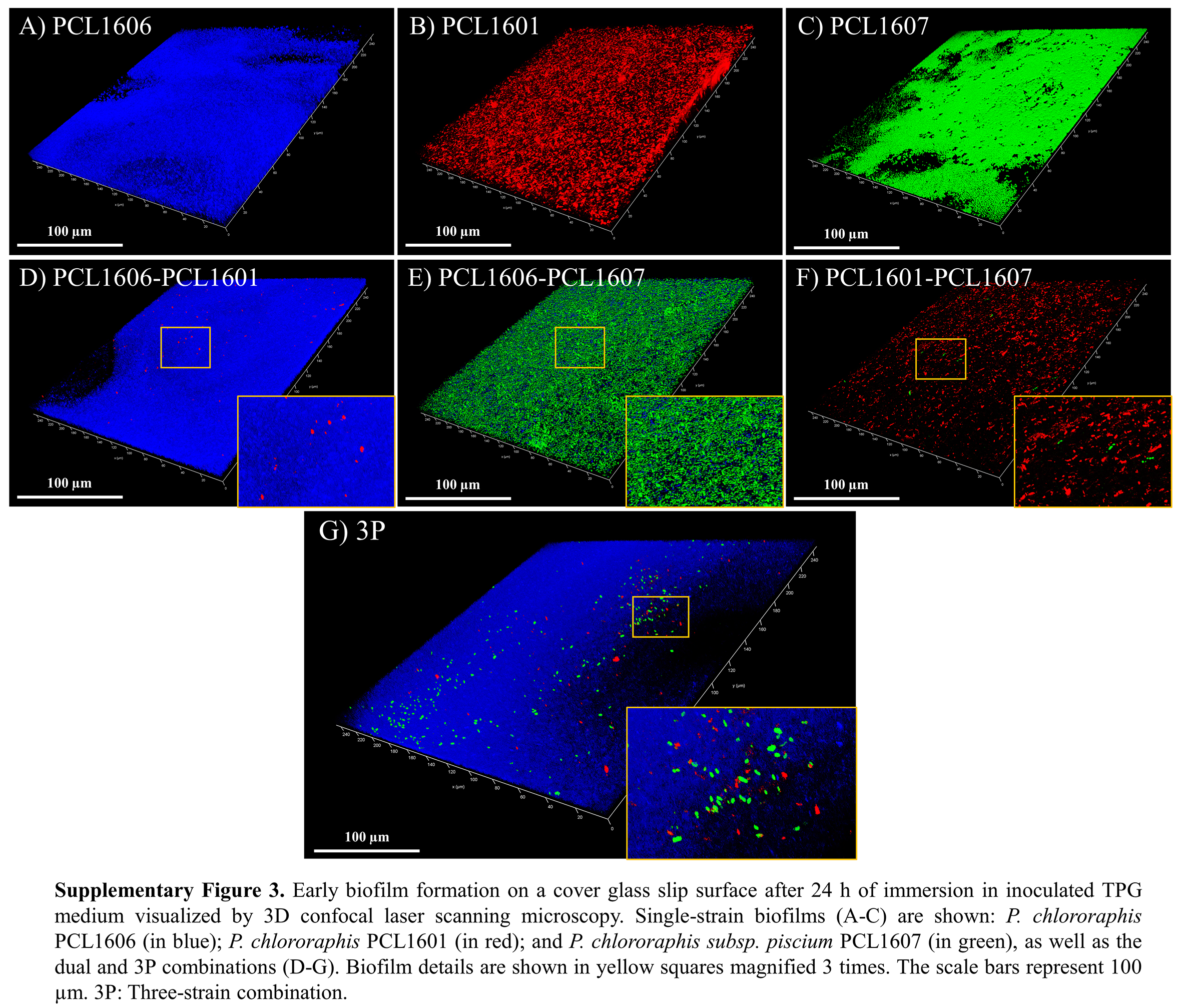

Supplement: Supplementary Figure 3 — Early biofilm formation on a cover glass slip surface after 24 h of immersion in inoculated TPG medium visualized by 3D confocal laser scanning microscopy. Single-strain biofilms (A–C) are shown: P. chlororaphis PCL1606 (in blue); P. chlororaphis PCL1601 (in red); and P. chlororaphis subsp. piscium PCL1607 (in green), as well as the dual and 3P combinations (D–G). Biofilm details are shown in yellow squares magnified 3 times. The scale bars represent 100 µm. 3P: Three-strain combination. [file Image_3.tif]

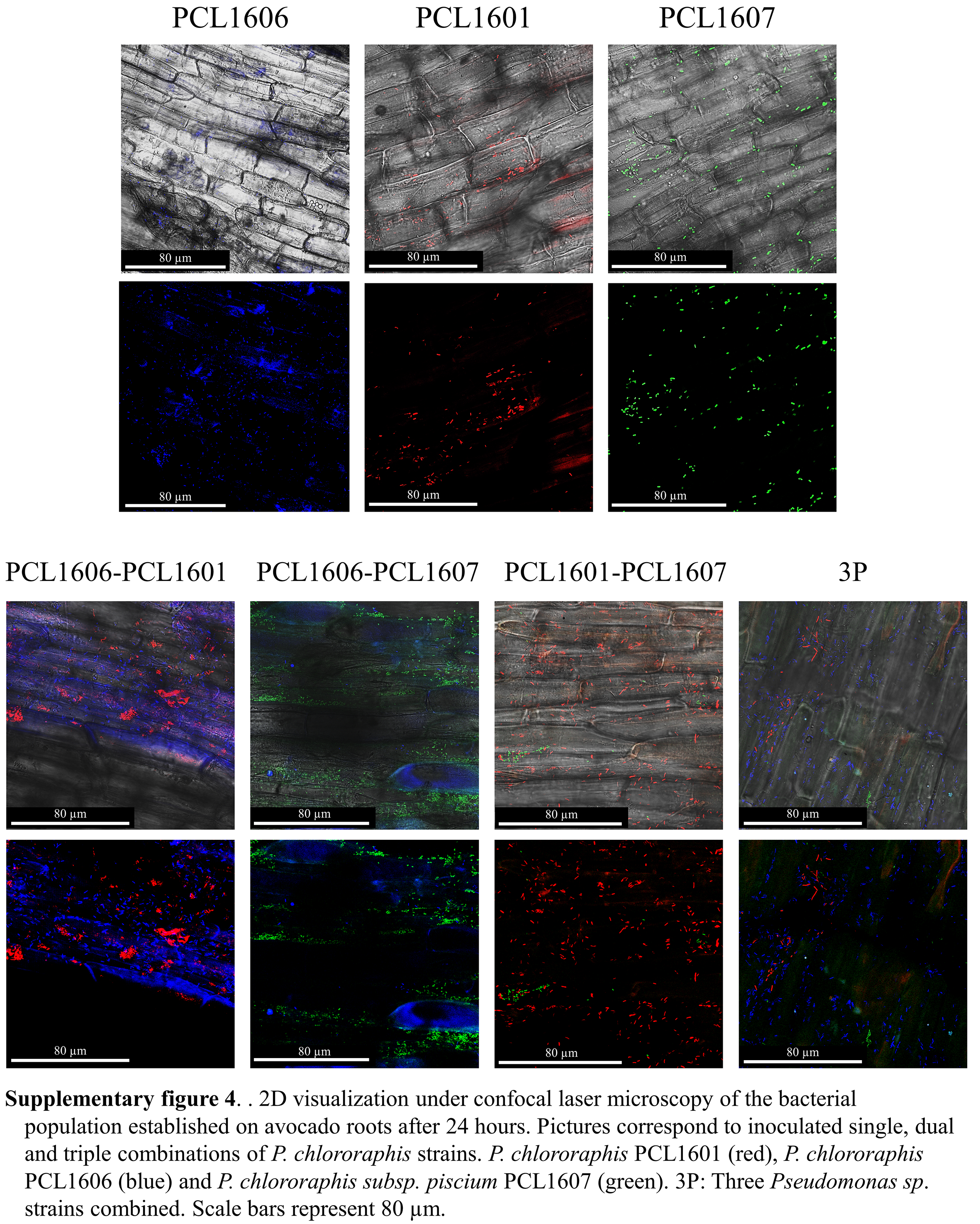

Supplement: Supplementary Figure 4 — 2D visualization under confocal laser microscopy of the bacterial population established on avocado roots after 24 hours. Pictures correspond to inoculated single, dual and triple combinations of P. chlororaphis strains. P. chlororaphis PCL1601 (red), P. chlororaphis PCL1606 (blue) and P. chlororaphis subsp. piscium PCL1607 (green). 3P: Three Pseudomonas sp. strains combined. Scale bars represent 80 µm. [file Image_4.tif]
