# Supplementary material for: Interplay between rhizospheric Pseudomonas chlororaphis strains lays the basis for beneficial bacterial consortia
Source: Front Plant Sci. 2022 Dec 15;13:1063182. doi: 10.3389/fpls.2022.1063182 (PMC9797978; doi:10.3389/fpls.2022.1063182)
Supplement: Supplementary file 5 [file Table_1.docx]

**Supplementary table 1**. Genomes available at Ezbiocloud web page, used for comparative genomics analysis.

| **Nº** | **Project accession** | **Taxon name** | **Strain name** |
| --- | --- | --- | --- |
| 1 | GCA_000264555.1 | *Pseudomonas chlororaphis subsp. aureofaciens* | O6 |
| 2 | GCA_000281915.1 | *Pseudomonas chlororaphis subsp. chlororaphis* | 30-84 |
| 3 | GCA_000698865.1 | *Pseudomonas chlororaphis subsp. aureofaciens* | PA23 |
| 4 | GCA_000963835.1 | *Pseudomonas chlororaphis* | PCL1606 |
| 5 | GCA_001921865.1 | *Pseudomonas sp.* | PCL1601 |
| 6 | GCA_003850365.1 | *Pseudomonas chlororaphis subsp. piscium* | ATCC 17809 |
| 7 | GCA_003850445.1 | *Pseudomonas chlororaphis subsp. piscium* | PCL1391 |
| 8 | GCA_003850465.1 | *Pseudomonas chlororaphis subsp. piscium* | PCL1607 |
| 9 | GCA_003850565.1 | *Pseudomonas sp.* | CMR12a(R) |
| 10 | GCA_003850585.1 | *Pseudomonas chlororaphis subsp. piscium* | ToZa7 |
| 11 | GCA_003851165.1 | *Pseudomonas chlororaphis subsp. aurantiaca* | M12 |
| 12 | GCA_003851205.1 | *Pseudomonas chlororaphis subsp. aurantiaca* | 449 |
| 13 | GCA_003851265.1 | *Pseudomonas chlororaphis subsp. aurantiaca* | M71 |
| 14 | GCA_003851345.1 | *Pseudomonas chlororaphis subsp. aurantiaca* | Q16 |
| 15 | GCA_003851405.1 | *Pseudomonas chlororaphis subsp. aureofaciens* | 66 |
| 16 | GCA_003851495.1 | *Pseudomonas synxantha* | 28887 |
| 17 | GCA_003851785.1 | *Pseudomonas chlororaphis subsp. aureofaciens* | Pb-St2 |
| 18 | GCA_003851835.1 | *Pseudomonas chlororaphis subsp. aurantiaca* | DSM 19603(T) |
| 19 | GCA_003851905.1 | *Pseudomonas chlororaphis subsp. aureofaciens* | DSM 6698(T) |
| 20 | GCA_003851925.1 | *Pseudomonas chlororaphis subsp. aureofaciens* | ChPhzTR39 |
